# Future career plans of Malawian medical students: a cross-sectional survey

**DOI:** 10.1186/1478-4491-10-29

**Published:** 2012-09-13

**Authors:** Kate L Mandeville, Tim Bartley, Mwapatsa Mipando

**Affiliations:** 1 Department of Global Health and Development, London School of Hygiene and Tropical Medicine, 15–17 Tavistock Place, London, WC1H 9SH, United Kingdom; 2 Department of Physics, Imperial College London, Exhibition Road, London, SW7 2AZ, United Kingdom; 3 Department of Physiology, Malawi College of Medicine, Chichiri, Blantyre 3, Malawi

**Keywords:** Human resources for health, Physicians, Medical education, Migration, Malawi

## Abstract

**Background:**

Malawi has one of the lowest physician densities in the world, at 1.1 doctors per 100,000 population. Undergraduate training of doctors at the national medical school has increased considerably in recent years with donor support. However, qualified doctors continue to leave the public sector in order to work or train abroad. We explored the postgraduate plans of current medical students, and the extent to which this is influenced by their background.

**Methods:**

A self-administered questionnaire was developed after discussion with students and senior staff. This included questions on background characteristics, education before medical school, and future career plans. This was distributed to all medical and premedical students on campus over 1 week and collected by an independent researcher. One reminder visit was made to each class. Chi-squared tests were performed to investigate the relationship of student characteristics with future career plans.

**Results:**

One hundred and forty-nine students completed the questionnaire out of a student body of 312, a response rate of 48%. When questioned on their plans for after graduation, 49.0% of students plan to stay in Malawi. However, 38.9% plan to leave Malawi immediately. Medical students who completed a ‘premedical’ foundation year at the medical school were significantly more likely to have immediate plans to stay in Malawi compared to those who completed A-levels, an advanced school-leaving qualification (*P* = 0.037). Current premedical students were slightly more likely to have immediate plans to work or train in Malawi compared to medical students (*P* = 0.049). However, a trend test across all the years was not significant. When asked about future plans, nearly half of students intend to work or train outside Malawi.

**Conclusions:**

The majority of respondents plan to leave Malawi in the future. The effectiveness of the substantial upscaling of medical education in Malawi may be diminished unless more medical students plan to work in Malawi after graduation.

## Background

Higher densities of health workers in a population are associated with improved health outcomes [1-4]. However, there is a critical shortage of health workers in Malawi, which is impeding the delivery of many health programs [5-8]. In 2004, the number of nurses and midwifes stood at 60 per 100,000 population, and doctors at only 2 per 100,000 [9]. Reasons include a chronic underproduction of trained personnel and out-migration, both from the public sector and the country as a whole [7].

**Figure 1:**
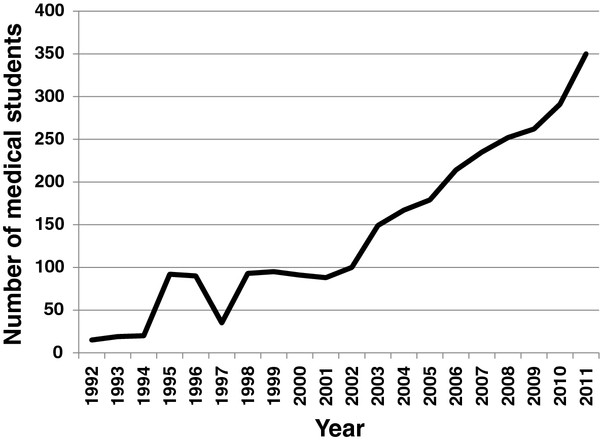
**Number of medical students enrolled at Malawi College of Medicine, 1992–2011**.

In 2004, the Malawi government introduced an Emergency Human Resources Plan (EHRP) [6,10,11]. This included retention measures such as a 52% salary supplement and incentives such as subsidized accommodation and transport. It also introduced a substantial increase in pre-service training of 11 cadres of health workers [12]. Enrolment at the national medical school has increased from 15 medical students when it opened in 1992 to 350 in 2011 (Figure 1). The EHRP was mostly funded by international donors, and is estimated to have cost approximately USD 96 million between 2004 and 2009, of which expanding training was the major component at USD 53 million [12].

A survey of graduates from the one medical school in Malawi was undertaken in 2006 [13]. Of the 254 graduates since the foundation of the College of Medicine (COM), 123 were working in Malawi. If those in private practice (*n* = 11) or working for non-governmental organizations (*n* = 2) are excluded, only 43% of graduates remain in the Malawian public sector. However, most of these graduates would have left Malawi before the new EHRP retention measures came into place.

The cost of training one doctor in Malawi has been estimated at USD 57,000 [14]. The EHRP scale-up of pre-service training will only be cost-effective if students intend to stay in Malawi after graduation. Current literature tends to focus on practicing health workers rather than those in training. With many countries implementing large increases in pre-service training to counter their health worker shortages, exploration and awareness of trainees’ postgraduate intentions may alert planners and policymakers to impending workforce issues. In this survey, we sought to investigate the postgraduate plans of current medical students at the COM in light of the improved conditions in the public sector. We also assessed the extent to which these plans are influenced by their background.

## Methods

### Setting

Malawi is a low-income country, with a gross national income of USD 810 per capita in 2008 [15]. Although densely populated for its size, 81.2% of the population still lives in rural areas [15]. Malawi has some of the worst health indicators in the region, with a maternal mortality ratio of 510 per 100,000 and an infant mortality rate of 65 per 1,000. Life expectancy at birth stands at 53 years and adult HIV prevalence is 11.2% [15].

The government spends 9.3% of total expenditure on health, with health service provision split between government facilities (60% of services) and faith-based organizations (40%) [12,15]. There are four tertiary hospitals based in the main urban areas of Lilongwe, Blantyre, Mzuzu, and Zomba. In rural areas, government district hospitals and mission hospitals supply secondary care, with health centers and health posts providing primary care [16]. The private sector in Malawi is small compared to other countries, with a handful of private hospitals and more numerous small private clinics.

There is one medical school in Malawi based in Blantyre, which was established in 1991 [17]. Admission to medical school is based primarily on academic achievement at secondary school. There are several types of secondary schools in Malawi, including private, government boarding (state-run, but fee-paying), government day (state-run, with nominal fees), and others (for example, seminaries or mission schools). Government boarding schools operate selective entry, based on a competitive examination at 11 years. There are three routes into medical school, all requiring a higher level of science education than that obtained through the basic school-leaving Malawian School Certificate of Education (MSCE). Firstly, candidates may take ‘A-levels’ in science subjects. A-levels are advanced qualifications obtained after 6 years of secondary education compared to the normal 4 years and are offered only in certain private schools in Malawi. Alternatively, candidates may complete 2 or 3 years of a Bachelors of Science degree at the University of Malawi and then transfer to the medical course. Lastly, for those competing for entry solely on the basis of their MSCE grades, they will need to complete a ‘premedical’ or foundation year which is an intensive year of science tuition to standardize knowledge with those who have taken a BSc or A-levels before entry.

The medical course itself lasts 5 years [17]. After qualification, graduate doctors complete an 18-month internship before registering with the Medical Council of Malawi [13]. Traditionally, those doctors wishing to pursue postgraduate specialization have trained abroad, with the consequence that many never returned [17]. CoM has now started 4-year Masters of Medicine (MMed) specialty training programs, where doctors work and train in the tertiary hospitals guided by the specialists there. Currently, they are only offered in certain specialties and most require a proportion of the training to be spent in South Africa [13]. Visa restrictions here prevent residence after the end of training, with most specialists hopefully returning to Malawi [13]. Other specialties require all training to be completed outside Malawi, as the expertise or facilities are not available in country.

### Study population

We aimed to include all medical students from Years 1 to 5. We also included students in the premedical year.

### Data collection

We developed a self-administered questionnaire ( Additional file 1) after discussion with senior tutors at the College and a focus group of five medical students from different years, selected through convenience sampling. The questionnaire was piloted on students from the focus group for clarity and understanding.

We obtained demographic information on each respondent, including district of origin, type of secondary school attended, and higher education prior to medical school. We asked about students’ intended specialty and their career plans immediately after graduation, differentiating between medical practice and postgraduate training in Malawi, in Africa, or elsewhere. As students may intend to complete their internship in Malawi and leave afterwards, we also asked about plans at some point in the future. Here, students could mark as many as applied, and the results are presented as percentages of responses rather than students.

The questionnaires were distributed to all students present on the campus of the College during one week in September 2008. One reminder visit was made to each class. The questionnaires were anonymous, and were distributed and collected by an independent researcher.

The College Registry provided data on trends in enrolment and background information on applicants for the 2009 academic year.

### Analysis

Pre-specified analyses were carried out using Chi-squared tests (or Fisher’s exact test where expected frequencies were less than 5). Stata-10 was used for all analysis.

We compared immediate plans against: gender, year of study, type of secondary school (government/private), and higher education. A Chi-squared test for trend for year of study against immediate plans to work or train in Malawi was performed. We also looked at whether the lack of specialist training within Malawi for a respondent’s intended specialty influenced their immediate plans to leave Malawi. Intended specialties were coded as to whether a training program was available or not available in Malawi. Respondents who had chosen two specialties that were available and not available were excluded. Finally, we investigated differences between respondents and non-respondents using Chi-squared tests.

## Results

**Figure 2:**
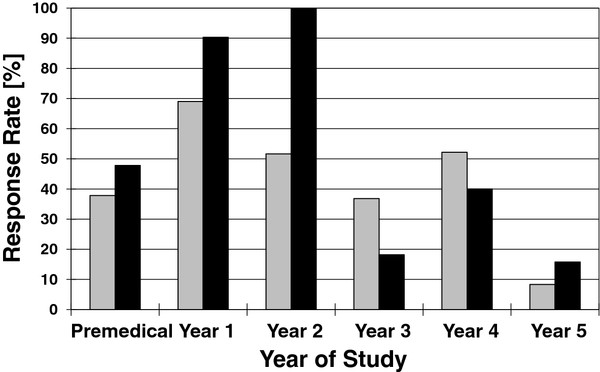
**Year of study and sex distribution of respondents.** Grey, male; Black, female.

One hundred and forty-nine students (59.9% men) responded to our survey out of a total student population of 312, a response rate of 48%. Whilst the response rate was better in the preclinical years (Years 1 and 2), it was much lower from those students in Years 3 to 5 (Figure 2). Non-respondents did not differ significantly from respondents by gender except in Years 1 and 2, where virtually all women in both years answered the survey.

### District of origin

**Figure 3:**
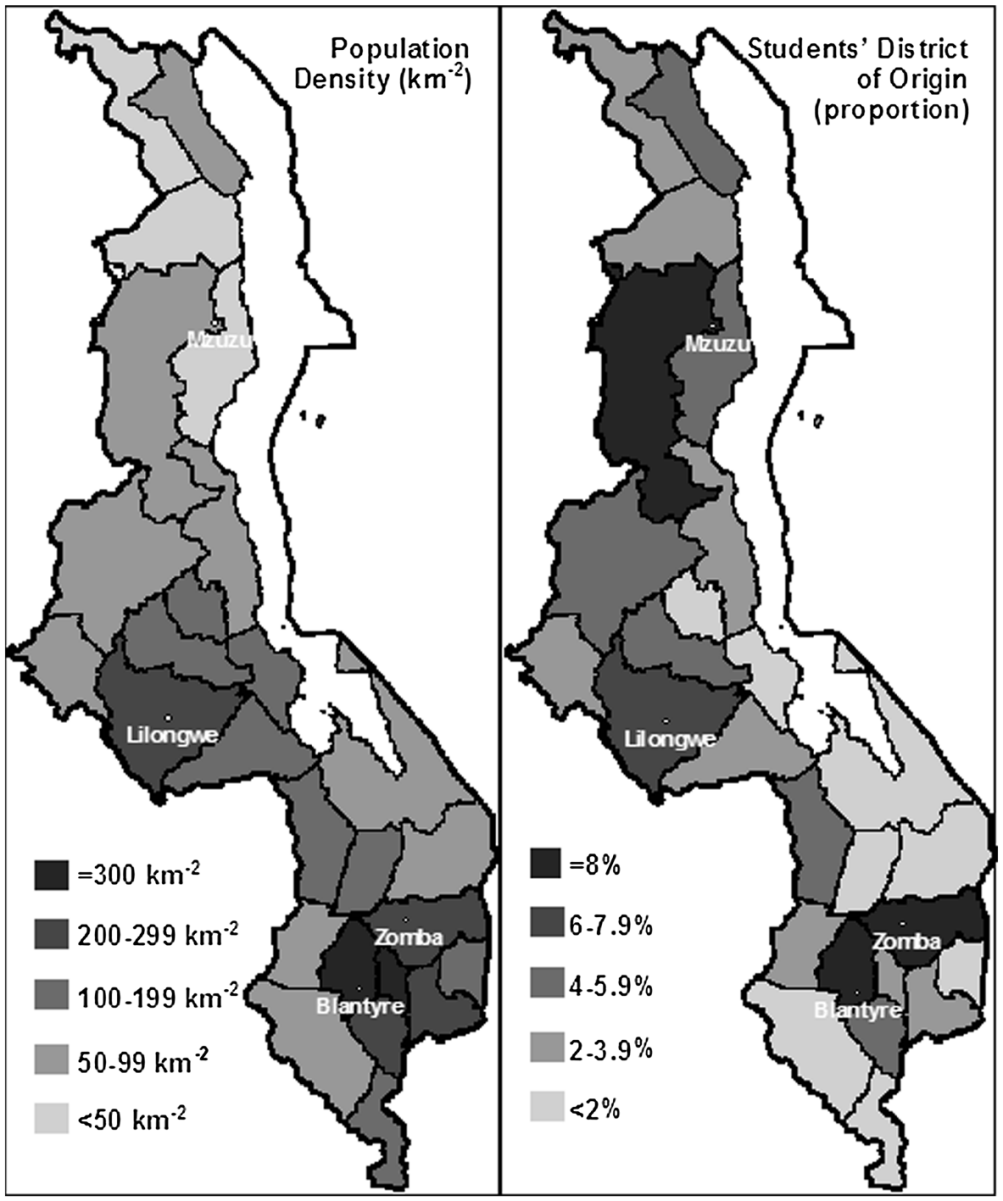
**Population density of Malawian districts compared to medical students’ district of origin.** Population data from the National Statistical Office of Malawi [19].

(Figure 3) compares the 2008 population densities for each district [18] to the percentage of students who indicated that district as their district of origin. The main urban areas of Blantyre, Lilongwe, Zomba, and Mzuzu have a higher population density and percentage of originating students. Overall, the rural districts in the south of the country appear to be under-represented in the student population compared to the northern districts.

### Secondary education

Most of our respondents (38.4%) had attended a private school for their secondary education, 27.4% government boarding schools, and only 5% government day schools (Figure 1).

**Figure 4:**
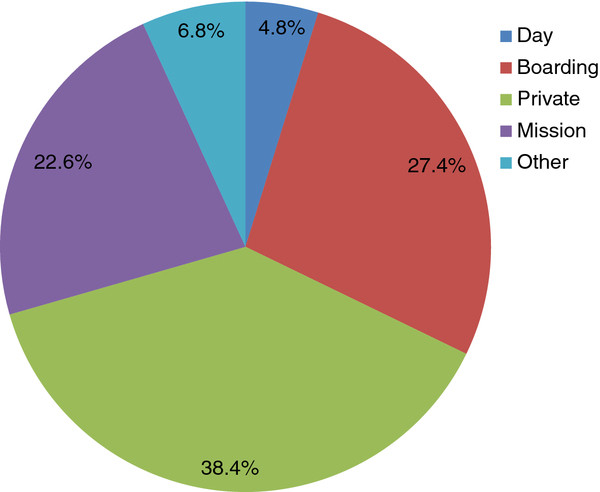
**Type of secondary school attended by medical students**.

### Higher education prior to medical school

The majority of students (73.8%) had completed the premedical course before entering medical school, 13.5% had completed A-levels and 11.1% had undertaken a BSc degree.

### Intended specialty

One hundred and twenty students (80.5% of total sample) indicated their intended specialty and 10 respondents saying that they were undecided (6.7%). The most popular specialties were: surgery (17.5%), cardiology (12.5%), pediatrics (12.5%), medicine (10.0%), obstetrics and gynecology (9.2%), and neurosurgery (8.3%). Overall, 44% of respondents to this question chose a specialty for which training is not currently available in Malawi.

### Future career plans

**Figure 5:**
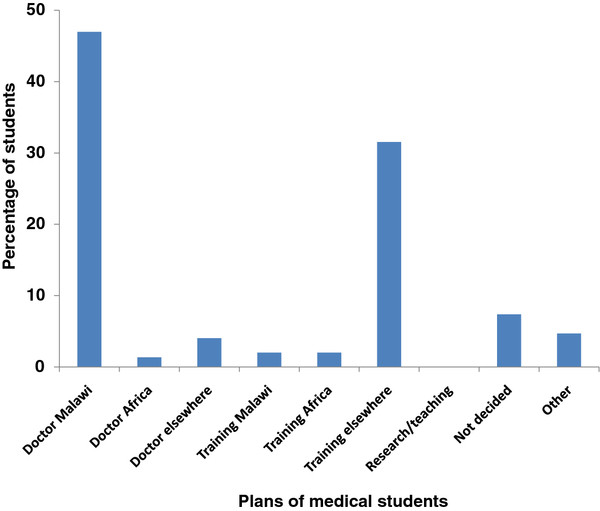
**Plans of medical students immediately after graduation**.

Nearly half of medical students intend to practice in Malawi immediately after graduation (47.0%), compared with 1.3% in Africa and 4.0% outside Africa. Whilst 2.0% of students are planning to train either in Malawi or in Africa, 31.5% intend to train outside Africa immediately after graduation (Figure 5).

If work and training options are combined, 49.0% of students plan to stay in Malawi immediately, but 38.9% will leave Malawi immediately.

**Figure 6:**
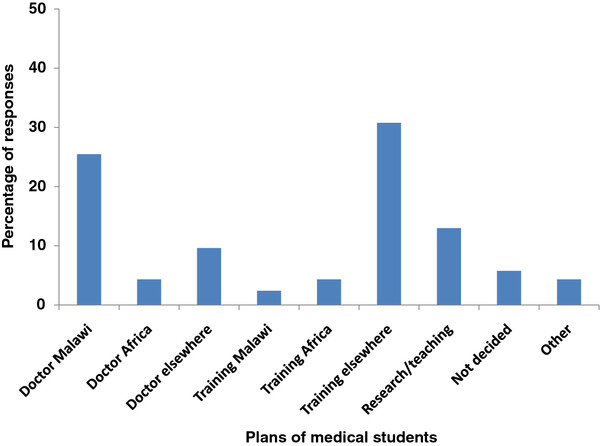
**Plans of medical students in the future**.

When asked about plans in the future (Figure 6), there is a higher percentage of responses for training elsewhere (30.8%) than practicing in Malawi (25.5%). Nearly half (49.0%) of responses are for options outside Malawi.

We performed selected analyses to investigate the relationship between selected aspects of students’ backgrounds and their future plans. We combined service and training options to give ‘Malawi’ or ‘Africa/Elsewhere’ outcomes as above. Medical students who completed the premedical year were significantly more likely to have immediate plans to stay in Malawi compared to those who completed A-levels (31.5% versus 3.4%, Fisher’s exact test, *P* = 0.037), but not BSc (31.5% versus 5.4%, *χ*^2^ = 0.147, *P* = 0.70). Current premedical students were more likely to have immediate plans to work or train in Malawi compared to medical students (*χ*^2^ = 3.84, *P* = 0.049).

A Chi-squared test for trend for year of study against immediate plans to work or train in Malawi was not significant (although numbers were small in the higher years and there may have been insufficient power to detect a trend). Analyses of gender and type of secondary school (government/private) against immediate plans were also not significant (*P* = 0.97 and *P* = 0.15, respectively). There was no association between the lack of availability of a training program for an intended specialty and immediate plans to leave Malawi.

## Discussion

Nearly 40% of medical students who responded to this survey are considering leaving Malawi after graduation and nearly half at some point in the future. One-third of respondents are currently planning to train outside Africa after graduation. Some of these students may return to Malawi after further training, although this has not been the norm in the past [13]. If all those who intend to leave do not return, then the financial loss for the government and external donors will be substantial [14].

These findings are in contrast to a survey of over 1000 medical students in Lusophone Africa [19], which found that the vast majority intended to work in their country in the future (Angola = 79.3%, Guinea-Bissau = 90.7%, and Mozambique = 80.2%). In addition, a survey of 51 final year Malawian healthcare students (including medical, nursing, and clinical officer students) as part of the EHRP evaluation, which found that 75% of these students did not see themselves seeking employment outside Malawi [12]. However, cadres such as clinical officers have lower chances of employment outside their country of training compared to doctors, which may help explain this discrepancy.

An important factor for medical students in particular may be that postgraduate training opportunities are still limited within Malawi, both in terms of capacity and government subsidy. As the specialty training program in Malawi becomes more established, awareness of and attitudes towards in-country training may change, especially as more specialists return from their period in South Africa [13]. Indeed, there was no relationship between the lack of in-country training available in the intended specialty and plans to leave Malawi, which suggests that medical students may not be as familiar with the Malawian postgraduate training programs or that it is still competing with outside programs for credibility.

The data on students’ backgrounds revealed that Southern districts appeared under-represented in the student population. Indeed, since this survey, the Ministry of Education has introduced a system of proportional selection for tertiary education places in order to ensure equitable distribution which is based partly on district population density. Although the majority of our respondents had attended private schools, this may be due to respondent bias as students from private schools may be more likely to complete questionnaires.

Medical students who had completed a premedical year were significantly more likely to intend to stay in Malawi after graduation compared to those who had done A-levels. However, this may be confounded by type of secondary education, with private school pupils more likely to do A-levels and also more likely to leave Malawi. Alternatively, it may be a function of the premedical education itself that increases motivation to practice in Malawi. As more students are entering medical school through the premedical route, this bodes well for future retention. Current premedical students were also more likely to stay or work in Malawi, however these students are likely to be substantially less exposed to working conditions in the health service than compared to the students in the clinical years of the medical course. These students may also be less confident to give responses that break with social expectations (for example, leaving Malawi), given their younger age and recent university entry.

This study indicates that there are bodies of students with differing intentions to work in Malawi after graduation. This offers some support to a policy of targeted student selection in order to enhance retention of doctors in Malawi, however more research would be needed to tease out those student characteristics associated with retention.

The internal validity of this study should be high. It was developed and piloted with the aid of medical students, which should increase face and construct validity. The questionnaires were self-administered, anonymous, and distributed by an independent researcher with no affiliation to the College. This would have minimized any socially desirable response or interviewer bias. External validity is likely to be low, however, due to the context-specific nature of medical education and the policy environment.

There are limitations to this study. First, these are stated intentions only. Students may end up acting very differently to their responses. For example, nearly as many students chose future specialties for which training is not yet available in Malawi as those for which training can be provided in-country. These intentions are likely to be influenced in the future by opportunities for funded scholarships and stronger personal reasons for staying in Malawi. However, in the absence of formal monitoring data for graduates, stated intentions may give an early indication of future workforce issues. In addition, medical students tend to be high-achievers, and as such may have given more thought to their career plans than their peers.

Our interpretation is limited by the much lower response rate in Years 3 to 5, where it was more difficult to access students as they were primarily based in hospitals, rather than the lecture format of earlier years, so questionnaire distribution was limited. Students in these years are likely to be different from preclinical students, as they will have had more exposure to clinical environments. This may make them even more likely to intend to leave Malawi after graduation due to their experience of poor working conditions and discussions with senior colleagues. Indeed, a survey which asked first year medical students and junior doctors in Nepal whether they intended to migrate to a developed country after graduation found that whilst 40.3% of students planned to migrate, this rose to 53% amongst junior doctors [20].

Alternatively, the converse may be true as clinical experiences may lead to strengthened commitment to service delivery in their country. Clinical students may also be more aware of the incentives introduced under the EHRP and therefore more likely to remain in Malawi. Any future surveys should make every effort for a high response rate from clinical years as well. There were significantly more female respondents in Years 1 and 2, however an analysis of immediate plans by gender showed no significant differences between men and women with regard to staying in or leaving Malawi. It may be that women are more conscientious at answering questionnaires than men at that stage.

Possible future work could investigate the differences in attitudes towards working in Malawi after the premedical year compared to students entering medical school with A-levels. Ideally, a cohort of students would be followed through medical school, allowing exploration of the factors and experiences which influence the intention to stay in Malawi. Qualitative work with graduates who have left Malawi for training elsewhere and reasons for their choice of destination would also be useful.

## Conclusions

The majority of respondents, who were concentrated in the preclinical years, intend to leave Malawi in the future. The effectiveness of the substantial upscaling of medical education in Malawi may be diminished unless more medical students plan to work in the Malawian public sector after graduation. Future work is needed to explore how students’ intentions change over the medical course.

## Abbreviations

COM: College of Medicine, University of Malawi; EHRP: Emergency Human Resources Programme.

## Competing interests

KLM set up and directs Medic to Medic, a program that sponsors disadvantaged medical students at the Malawi College of Medicine (http://www.medictomedic.org.uk).

## Authors’ contributions

KLM designed the study, performed the data analysis, and wrote the manuscript. TB designed the questionnaire and carried out the data collection. MM helped with the data collection and the manuscript preparation. All authors modified and approved the final manuscript.

## Supplementary Material

Additional file 1 This is the study questionnaire investigating the background and future plans of medical students at the Malawi College of Medicine. Click here for file
