# Supplementary material for: Future career plans of Malawian medical students: a cross-sectional survey
Source: Hum Resour Health. 2012 Sep 13;10:29. doi: 10.1186/1478-4491-10-29 (PMC3465242; doi:10.1186/1478-4491-10-29)
Supplement: Additional file 1 — This is the study questionnaire investigating the background and future plans of medical students at the Malawi College of Medicine. [file 1478-4491-10-29-S1.doc]

**Medical training in Malawi: Questionnaire**

This is a survey which is looking at the background and future plans of current medical students at the Malawi College of Medicine.

Please be honest; all answers will be treated with the strictest confidence and will not be seen by any teaching staff.

## **Part A: About yourself**

| Age |  |
| --- | --- |
| Sex (*please tick* 🗹*)* | 🞎 Male 🞎 Female |
| Year of study (*please tick* 🗹*)* | 🞎 Premed 🞎1 🞎2 🞎3 🞎4 🞎5 |
| District of origin *(e.g. Dowa)* |  |
| Town/Village of origin *(e.g. Mponela)* |  |

## **Part B: Your education prior to Medical College**

| What **type** of secondary school did you study for your MSCE exams? *(please tick* 🗹*)* | 🞎 Community day school  🞎 Government boarding school  🞎 Private school  🞎 Mission school/seminary  🞎 Other *(please specify):* ___________ |
| --- | --- |
| How old were you when you **started** secondary school? |  |
| How old were you when you **finished** secondary school? |  |
| What did you study immediately **before** entering medical college? *(Please tick* 🗹*)* | 🞎 Premedical course  🞎 A-Levels  🞎 BSc degree  🞎 Other *(please specify):* ___________ |

## **Part C: Your background**

| What does your father do for work? *(e.g. Teacher)* | *Please specify:*  ____________________________________  Or 🞎 does not work 🞎 deceased |
| --- | --- |
| What does your mother do for work? *(e.g. Nurse)* | *Please specify:*  ____________________________________  Or 🞎 does not work 🞎 deceased |
| How many children are in your family? |  |

**Part D: Your future plans**

| What is your intended speciality? *(e.g. Paediatrics)* |  |
| --- | --- |
| What do you intend to do **immediately** after graduation? *(please tick* 🗹 *ONE)* | 🞎 Doctor in Malawi  🞎 Doctor abroad 🞎 in Africa  🞎 elsewhere  🞎 Specialist/postgraduate training 🞎 in Malawi  🞎 in Africa  🞎 elsewhere  🞎 Research/Teaching  🞎 Not yet decided  🞎 Other *(please specify):* _________________ |
| What do you intend to do **at some point** in the future? *(please tick* 🗹 *all that apply)* | 🞎 Doctor in Malawi  🞎 Doctor abroad 🞎 in Africa  🞎 elsewhere  🞎 Postgraduate training 🞎 in Malawi  🞎 in Africa  🞎 elsewhere  🞎 Research/Teaching  🞎 Not yet decided  🞎 Other *(please specify):* _________________ |
| What kind of incentives would encourage you to work in rural areas of Malawi? *(please tick* 🗹 *TWO that are most important to you)* | 🞎 Higher salary  🞎 Opportunities for career progression  🞎 Time-limited period  🞎 Loan for a car  🞎 Accommodation provided  🞎 Other *(please specify):* _________________ |
| For what reasons would you consider moving abroad, either in Africa or elsewhere in the world? *(please tick* 🗹 *TWO that are most important to you)* | 🞎 Higher salary  🞎 Career opportunities/postgraduate training  🞎 Family reliance/expectance on income  🞎 Other *(please specify):* _________________ |
| How do you feel about bonding students to work in rural areas for a set period once they graduate? *(please tick* 🗹 *as appropriate: 1= strongly in favour…5 = strongly against)* | 🞎1 🞎2 🞎3 🞎4 🞎5 |

Thank you very much for taking the time to fill in this questionnaire.
